# Scientific Items

**Published:** 1882-07-20

**Authors:** 


### SCIENTIFIC ITEMS.

**There** is apparently no limit to the activity of American inventors. During the first five months of the present year, there were filed at the Patent Office 13,244 applications for patents, being 2,307 more than for the same period in 1881, and 3,039 more than for the corresponding months of 1880. The receipts of the office for the same time were $428,805.65, being an increase of $64,623.55 over 1881, and $90,660.85 over 1880. The total receipts for the present year, should the rate here shown be maintained, will be over one million dollars, and the number of applications more than 36,000. Of this multitude of inventions, covering every field in which human intelligence is at work, a large proportion, of course, will be of questionable or trifling utility. But a respectable percentage, at least, are of genuine value, and here and there in the list will be found such fruits of genius and patient labor as become landmarks in the history of the world's progress. One has only to reflect upon the astonishing number of these yearly contributions to the resources of science and art, to realize in what a pregnant age we are living, and how swift is the march of civilization toward a future of which the most sober estimates seem like the play of an extravagant fancy.—*Mechanical News.*

**A Demonstration of a New Method.**—Some time ago a most interesting demonstration of a new method of preserving meat was given at York terrace, Regent's Park, England. Instead of steeping the meat in an antiseptic, the preservative compound was introduced into the veins of the living animal, and from them into every part of the body. The sheep upon which the experiment was performed was first stunned by a blow on the head given with a wooden mallet. The left jugular vein was then laid bare, a pint of blood drawn off and about two pints of the preservative, dissolved in warm water and kept at blood heat, was injected into the opened vein. The sheep was then killed in the ordinary way, the whole occupation not occupying more than four minutes. The antiseptic used in this experiment was boracic acid, which did not in the slightest degree affect the quality or flavor of the meat, while the results of later experiments show that meat so treated will keep perfectly good without the use of ice or refrigerators for five or six weeks in summer and two or three months in cold weather.—*New York World.*

**There** is something stimulating to the imaginatiou in the estimate of two million horse-power as the useful capacity of the American Fall at Niagara—in the ponderous machinery which it is proposed to erect on the brink of the falls for converting this power into electric energy—and in the 10,000 miles of copper cable by which this force is to be distributed to sixty-five leading American cities and used by them for heating, illuminating and general industrial purposes. The scale on which this enterprise is projected ought to satisfy the most ardent enthusiast; and the statement has been made so many times that the author of the scheme has bought the twelve acres known as Prospect Park, as a preliminary step to getting his Archimedean lever in position, that it would be hardened skepticism to disbelieve it any longer. But the unpleasant reflection arises that the work of getting the project on paper, which is all that has yet been done, is at once the easiest and most inspiring part of the job. The prosaic details of the actual performance are yet to come, and will be much more tedious than the bold sketching of the outline. If nothing more is intended, however, than to create a company, and induce a sufficient number of credulous capitalists to put their good money into the undertaking, after the fashion of the Keely Monor stockholders, there are no mechanical obstacles to be overcome, and the Niagara genius has smooth sailing before him.—*Mech. News.*

**A New Theory of the Formation of Coal.**—After a protracted microscopic study of coal, Prof. Reinsch has come to the conclusion that coal was not derived from land plants, but chiefly from microscopic forms of "a lower order of protoplasm." He holds that plants of a higher order have contributed but a fraction of the mass of coal veins, however numerous they may have been in some instances. In a recent lecture, stating his conclusions, Prof. Reinsch referred to the fact that Dr. Muck, of Bochum, held that algæ have mainly contributed to the formation of coal, and that marine plants were rarely found in coal because of their tendency to decompose, and that calcareous remains of molusks disappeared on account of the rapid formation of carbonic acid during the process of carbonization.—*Independent Practitioner.*

**Metalic Writing Pencils.**—These pencils consist, according to the Industrieblatter, of an alloy of lead, bismuth and quicksilver. The ingredients vary according to desired hardness of the pencils. The ordinary proportions are: Lead, 70; bismuth, 90, and quicksilver, 8 parts by weight. A larger proportion of lead and quicksilver makes the pencil softer, and produces darker marks in writing. The lead and bismuth are melted together and allowed somewhat to cool, when the quicksilver is added and the composition cast in proper moulds.—*Druggists Circular.*

**Baptiste-Jacob, the New Siamese Twins.**—The brothers Tocci, born in Turin in 1877, are considered to be even more curious than the famous Siamese twins. They have two well formed heads, two pairs of arms and two thoraces, with all internal organs; but at the level of the sixth rib they coalesce into one body. —*Ex.*

A RECENT German improvement in grain-cleaning machines consists in making the casings of stoneware made of ferruginous clay. The advantages claimed by the inventor for this covering over metal are that it lasts longer and works better, as the working-surface will not wear smooth.—*Ex.*

